# Steroid Resistant CD8^+^CD28^null^ NKT-Like Pro-inflammatory Cytotoxic Cells in Chronic Obstructive Pulmonary Disease

**DOI:** 10.3389/fimmu.2016.00617

**Published:** 2016-12-19

**Authors:** Greg Hodge, Sandra Hodge

**Affiliations:** ^1^Chronic Inflammatory Lung Disease Research Laboratory, Lung Research Unit, Hanson Institute, Adelaide, SA, Australia; ^2^Department of Thoracic Medicine, Royal Adelaide Hospital, Adelaide, SA, Australia; ^3^Department of Medicine, University of Adelaide, Adelaide, SA, Australia

**Keywords:** CD8^+^ NKT-like cell, steroid resistance, chronic obstructive pulmonary disease, CD28, IFNγ and TNFα, Pgp, HDAC2, Hsp90

## Abstract

Corticosteroid resistance is a major barrier to effective treatment in chronic obstructive pulmonary disease (COPD), and failure to suppress systemic inflammation in these patients may result in increased comorbidity. Although much of the research to date has focused on the role of macrophages and neutrophils involved in inflammation in the airways in COPD, recent evidence suggests that CD8^+^ T cells may be central regulators of the inflammatory network in this disease. CD8^+^ cytotoxic pro-inflammatory T cells have been shown to be increased in the peripheral blood and airways in patients with COPD, whereas smokers that have not progressed to COPD only show an increase in the lungs. Although the mechanisms underlying steroid resistance in these lymphocytes is largely unknown, new research has identified a role for cytotoxic pro-inflammatory CD8^+^ T-cells and CD8^+^ natural killer T-like (NKT-like) cells. Increased numbers of these cells and their significant loss of the co-stimulatory molecule CD28 have been shown in COPD, consistent with findings in the elderly and in clinical conditions involving chronic activation of the immune system. In COPD, these senescent cells expressed increased levels of the cytotoxic mediators, perforin and granzyme b, and the pro-inflammatory cytokines, IFNγ and TNFα. They also demonstrated increased cytotoxicity toward lung epithelial cells and importantly were resistant to immunosuppression by corticosteroids compared with their CD28^+^ counterparts. Further research has shown these cells evade the immunosuppressive effects of steroids *via* multiple mechanisms. This mini review will focus on cytotoxic pro-inflammatory CD8^+^CD28^null^ NKT-like cells involved in COPD and novel approaches to reverse steroid resistance in these cells.

## CD8^+^ Natural Killer T-Like (NKT-Like) Cells in Chronic Obstructive Pulmonary Disease (COPD)

**Figure 1:**
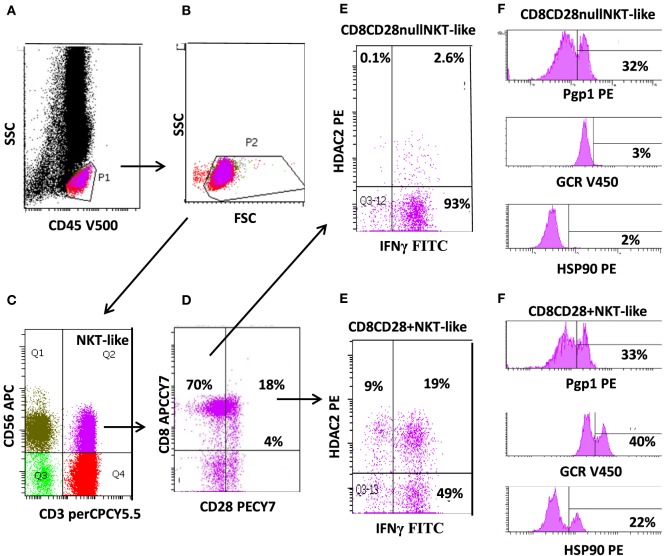
**Flow cytometric gating technique used to identify CD8^+^CD28^null^ natural killer T-like (NKT-like) cells (and CD8^+^CD28^+^ NKT-like cells) from the peripheral blood of patients with chronic obstructive pulmonary disease**. **(A)** Identification of lymphocytes as CD45^+^ low side scatter (SSC) events. **(B)** Removal of red blood cell contaminations removed by lymphocyte gating using forward scatter vs. SSC characteristics. **(C)** Identification of NKT-like cells as CD3^+^CD56^+^ events. **(D)** Identification of CD28^null^ and CD28^+^ NKT-like cells using CD8 vs. CD28 staining. **(E)** Expression of IFNγ and histone deacetylase (HDAC)2 in CD8^+^CD28^null^ and CD8^+^CD28^+^ cells. **(F)** Expression of P-glycoprotein-1 (Pgp1), glucocorticoid receptor (GCR), and heat shock protein (Hsp)90 expression in CD8^+^CD28^null^ and CD8^+^CD28^+^ cells. Note: the majority of NKT-like cells are CD8^+^ and CD28^null^. These cells express reduced HDAC2, GCR, and Hsp90 but increased IFNγ compared with CD8^+^CD28^+^ NKT-like cells (Pgp1 unchanged).

Natural killer T-like cells comprise a unique subgroup of lymphocytes that express features of both T cells and natural killer (NK) cells. NKT-like cells co-express T-cell receptors and CD4 or CD8 (or CD4^−^/CD8^−^), together with markers associated with NK cells, such as CD56 (Figure 1C) and/or CD16 or CD161. Acquisition of CD11b represents an early event in CD8^+^ T-cell differentiation, which may allow extravasation to peripheral tissues (1, 2). These cells are a small but important subset of lymphocytes that represent a bridge between innate and adaptive immunity.

There has been conflicting evidence regarding changes in NKT-like cell numbers in COPD. Numbers of these cells have been reported to be decreased in the peripheral blood of patients with COPD (3). One study showed numbers to be unchanged (4), while a third reported increased numbers (5). However, further characterization into CD4^+^ or CD8^+^ NKT-like cells was not performed in any of these reports. NKT-like cells have also been reported to be increased in induced sputum and bronchoalveolar lavage (BAL) of COPD patients and, importantly, have been shown to be cytotoxic to autologous lung cells (3, 4, 6).

## Loss of CD28 on Senescent Lymphocytes in COPD

Following persistent antigenic stimulation, NKT-like cells can lose co-stimulatory molecules, undergo telomere shortening, and exhibit defective IL-2 production; changes that define the state of replicative senescence. The majority of these “effector senescent” lymphocytes are CD8^+^, CD45RA^+^, CD11a^bright^, CD28^null^ (Figure 1D), CD62L^−^, and CCR7^−^. Expansion of these cells are found in the elderly and in other clinical conditions involving chronic activation of the immune system such as viral infections, rheumatic, and autoimmune diseases (7). Increased numbers have also been reported in chronic inflammatory lung diseases including COPD and in patients following lung transplantation (8, 9).

## Steroid Resistance in CD8^+^CD28^null^ NKT-Like Cells in COPD

### Steroid Resistant CD8^+^ T Cells in COPD

Patients with COPD have been shown to be resistant to the immunosuppressant effects or glucocorticoids (10). Most of the investigations into steroid resistance in this disease have focused on the role of the airway macrophages and neutrophils (10); however, the mechanisms underlying steroid resistance in lymphocytes in patients with COPD until recently has been largely unknown. The role of T-cells is likely to be important in this regard, as their increased numbers have been reported in the lungs of patients with COPD. A study by Maeno et al. demonstrated an important requirement for CD8^+^ T cells in the development of cigarette smoke-induced emphysema. They suggested a unifying pathway whereby CD8^+^ T cells are the central regulators of the inflammation network in COPD (11). Inhaled corticosteroids have been shown to reduce exacerbation rates and improve health status in patients with COPD but can also increase the risk of pneumonia (12, 13). The numbers of bronchial CD8^+^ T-cells were reduced following long-term treatment with inhaled corticosteroids in ex-smoker COPD patients only but not persistent COPD smokers (12, 13).

There have been reports of increased numbers of CD8^+^ T cells in the peripheral blood, BAL, and lung parenchyma from COPD smoker and ex-smoker patients compared with healthy smokers and control subjects (14, 15). This indicates the systemic involvement of these cells in COPD. The production of the pro-inflammatory cytokines, IFNγ and TNFα, by CD8^+^ T cells was increased from peripheral blood, BAL, and intraepithelial compartments in patients with COPD. This was regardless of whether patients were receiving inhaled corticosteroids (14) indicating the lack of effectiveness of steroids at reducing pro-inflammatory cytokines by these cells. However, further lymphocyte subtyping with NKT-like cell markers was not performed. Steroid resistance was further shown *in vitro* by assessing the production of IFNγ by follicular CD8^+^ T cells in the presence of 0.1–1µM dexamethasome (16), although further subtyping of NKT-like subsets was not performed in the study. Recently, steroid resistant CD8^+^CD28^null^ NKT-like cells were reported to be increased in number and to express increased levels of the cytotoxic mediators, perforin and granzyme b. Pro-inflammatory cytokines, IFNγ and TNFα (8), were also increased in the peripheral blood of patients with COPD, confirming the important role of these lymphocytes in steroid resistance.

### P-glycoprotein-1 (Pgp1) in CD8^+^CD28^null^ NKT-Like Cells

P-glycoprotein is a transmembrane efflux pump well-characterized in drug-resistant cancer cells (17) and also thought to play a role in the function of steroid resistant lymphocytes in COPD. Pgp1 expression has been shown to be increased in T, NKT, and NK cells that also co-express IFNγ, TNFα, and granzyme b, in peripheral blood from COPD patients compared with healthy controls (Figure 1). However, further differentiation of NKT-like cells into CD4^+^ and CD8^+^ subsets was not performed (18).

**Figure 2:**
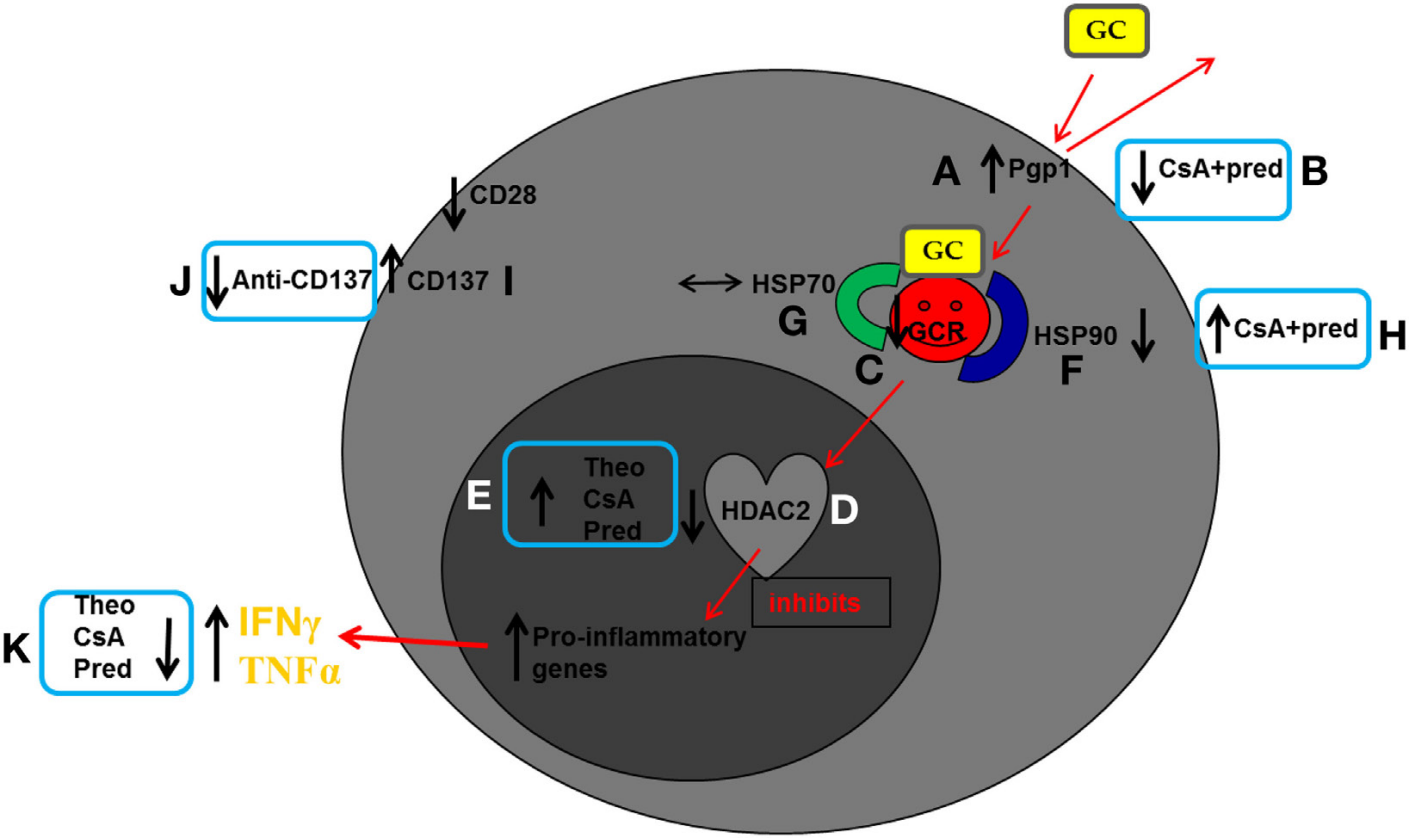
**Schematic diagram summarizing reported findings in peripheral blood CD8^+^CD28^null^ natural killer T-like (NKT-like) cells in chronic obstructive pulmonary disease (COPD)**. Glucocorticoids enter cells by overcoming membrane drug efflux pump P-glycoprotein-1 (Pgp1) and binding to the glucocorticoid receptor (GCR) in the cytoplasm. GCR must be bound to the molecular chaperones heat shock protein (Hsp)70 and Hsp90 to acquire a high-affinity steroid binding conformation, and traffic to the nucleus where engagement of histone deacetylases (HDACs), particularly HDAC2, results in reduction of pro-inflammatory gene activation. In COPD compared with age-matched healthy control subjects: **(A)** Pgp1^+^ NKT-like cells are increased in COPD, reducing intracellular levels of GC. Expression of GCR **(C)**, Hsp90 **(F)**, and HDAC2 **(D)** are decreased in CD8^+^CD28^null^ NKT-like cells (no change in Hsp70) **(G)** reducing steroid effectiveness. **(I)** The percentage of steroid resistant CD8^+^CD28^null^CD137^+^ NKT-like cells is increased. Possible therapeutic targeting to overcome steroid resistance CD8^+^CD28^null^ NKT-like cells in COPD: **(B)** Pgp1 is synergistically decreased in the presence of 2.5 ng/ml cyclosporine A (CsA) and 1µM prednisolone (pred). **(H)** Hsp90 expression is increased in the presence of 2.5 ng/ml CsA and 1µM pred. **(E)** HDAC2 expression is increased in the presence of 5 mg/ml theophylline, 2.5 ng/ml CsA, and 1µM pred. **(J)** Blocking CD137 expression with anti-CD137 antibody. **(K)** This targeting results in decreased IFNγ and TNFα pro-inflammatory cytokine expression.

Recent further investigations by the same authors comparing COPD patients with healthy age-matched controls showed no difference in Pgp1 expression between CD8^+^CD28^null^ NKT-like and CD28^+^CD8^+^ NKT-like subsets. However, the percentages of CD8^+^Pgp1^+^CD28^null^ NKT-like and CD8^+^Pgp1^+^CD28^+^ NKT-like cells were both increased in the COPD group (8) (Figure 2A). Treatment with very low-dose cyclosporine A (CsA), a Pgp1 inhibitor (2.5 ng/ml; approximately 25 times less than that used for transplant rejection therapy), combined with standard dose corticosteroid [1µM prednisolone (pred)] resulted in synergistic inhibition of pro-inflammatory cytokines in CD8^+^Pgp1^+^CD28^null^ NKT-like cells (18) (Figure 2B). These data indicate that these agents may be an effective add-on therapy to standard steroid treatment.

### Loss of Glucocorticoid Receptor (GCR) in CD8^+^CD28^null^ NKT-Like Cells in COPD

Glucocorticoids must bind to the GCR in the cytoplasm of a cell before being transported to the nucleus. A recent study examined the expression of GCR in pro-inflammatory NKT-like cells in the peripheral blood of patients with COPD (8). COPD was associated with increased percentage of CD28^null^ NKT-like cells compared with healthy controls. Loss of CD28 was associated with an increase in percentage of NKT-like cells producing IFNγ and TNFα and importantly, with a loss of GCR (8) (Figure 2C). A significant loss of GCR in CD8^+^CD28^null^ NKT-like cells was noted in both COPD patients and controls compared with CD8^+^CD28^+^ NKT-like cells (mean ± SEM: 9 ± 4% CD8^+^GCR^+^CD28^null^ NKT-like cells vs. 39 ± 7% CD8^+^GCR^+^CD28^+^ NKT-like cells in COPD). There was a significant correlation between GCR expression and IFNγ and TNFα production by CD8^+^ NKT-like cells. Taken together, these data show a loss of GCR in senescent CD8^+^CD28^null^ NKT-like cells and suggest that alternate treatment options to glucocorticoids are required to suppress pro-inflammatory cytokine production in patients with COPD.

### Decreased Histone Deacetylase (HDAC)2 in CD8^+^CD28^null^ NKT-Like Cells in COPD

Histone acetyltransferases and HDAC are enzymes that upregulate and downregulate pro-inflammatory gene transcription, respectively. HDAC2 is required by corticosteroids to switch off activated inflammatory genes and is reduced in lung macrophages in COPD (10). A recent study showed that HDAC2 expression was suppressed in pro-inflammatory CD8^+^CD28^null^ NKT-like cells in patients with COPD (19) and negatively correlated with the percentage of CD8^+^CD28^null^ NKT-like cells producing IFNγ or TNFα in all subjects (e.g., COPD: *R* = −0.789, *p* < 0.001 for CD8^+^CD28^null^ NKT-like cells producing IFNγ) (Figure 2D). Theophylline is an activator of HDAC and enhances the anti-inflammatory effects of corticosteroids in alveolar macrophages in COPD patients (20). Addition of theophylline has recently been shown to increase the anti-inflammatory effects of steroids in senescent lymphocytes from COPD patients (18). Addition of low-dose theophylline (5 mg/l) induced a synergistic upregulation of HDAC2 in CD8^+^CD28^null^ NKT-like cells in the presence of 1µM pred and 2.5 ng/ml CsA (Figure 2E). This was associated with a decrease in pro-inflammatory cytokine production by these cells. These findings suggest this form of therapy may enhance the anti-inflammatory effects of steroids and thus reduce inflammation caused by these cells in COPD.

### Decreased Heat Shock Protein (Hsp)90 in CD8^+^CD28^null^ NKT-Like Cells in COPD

Glucocorticoid receptor must be bound to molecular chaperones Hsp70 and Hsp90 to acquire a high-affinity steroid binding conformation and traffic to the nucleus (21). A recent study examined expression of Hsp70/90 in CD8^+^CD28^null^ NKT-like cells from the peripheral blood of patients with COPD (22). Loss of expression of Hsp90 and GCR from the CD8^+^CD28^null^ NKT-like cells in COPD was noted (Figure 2F), whereas expression of Hsp70 was unchanged (Figure 2G). The loss of Hsp90 was shown to correlate with the cytotoxic/pro-inflammatory potential of these cells and importantly, degree of airflow limitation in patients with COPD. The immunosuppressant, CsA, binds to the GCR–Hsp90 complex, but not Hsp70 (23), and was shown to upregulate Hsp90 with an associated decrease in pro-inflammatory cytokine production by CD8^+^CD28^null^ NKT-like cells when combined with 1µM pred (Figure 2H). The concentration of CsA (2.5 ng/ml) used in these *in vitro* experiments was 50 times less than that used for patients following lung transplant to prevent graft rejection. Hence, these low concentrations are not likely to be associated with any of the side effects reported with higher doses of this drug.

## Inhibiting CD137 Expression in CD8^+^CD28^null^ NKT-Like Cells in COPD

The loss of CD28 on CD8^+^CD28^null^ NKT-like cells from COPD subjects has been reported to be associated with an upregulation of the “alternate” co-stimulatory molecule CD137 (4-1BB) (24) (Figure 2I). Targeting CD137 has been shown to be effective in treatment of rheumatoid arthritis and may thus be effective in other diseases associated with increased expression of this co-stimulatory molecule, including COPD (25). *In vitro* studies showed that blocking CD137 with an anti-CD137 antibody following PHA stimulation of PBMC from COPD patients resulted in a decrease in the percentage of CD8^+^CD28^null^ NKT-like cells producing IFNγ, TNFα, and granzyme b production (26) compared with CD8^+^CD28^+^ NKT-like cells (Figure 2J), whereas stimulatory CD137 antibody increased production of these molecules. This indicates that targeting CD137 with anti-CD137 antibody may have novel therapeutic options for reducing inflammation in patients with COPD.

## Does Oxidative Stress Play a Role in Steroid Resistance in NKT-Like Cells?

There is increasing evidence that oxidative stress is important in the pathogenesis of COPD (27, 28). Oxidative stress occurs due to an increase of reactive oxygen species (ROS) causing damage to lipids, proteins, and DNA. Increased burden of oxidants from cigarette smoke and air pollutants and from ROS and reactive nitrogen species (RNS) released from inflammatory neutrophils, eosinophils, macrophages, and epithelial cells occurs in the lungs of COPD patients (27–29). The aging process is associated with a decrease in the antioxidant defense mechanisms in the lung resulting in increased ROS and RNS (30). Although there is a causal link between ROS, COPD, and aging in cellular senescence in many cells in the lung, sensitivity of individual lymphocyte subsets to oxidative stress and how this process affects disease progression remains largely unknown (30). While one study showed an association between ROS and cellular senescence in lymphocytes, some markers of oxidative stress were decreased (31). Increasing concentrations of ROS has been shown to suppress Th1 cells and increase Th2 cells, findings at odds with ours and many others in patients with COPD (30). Furthermore, it has been shown that neutrophils in the inflamed lung produce large amounts of ROS, which suppress T cells, while macrophages secrete cysteine and thioredoxin, which increase oxidation resistance of T cells (32). Although oxidative stress has been shown to inhibit expression of GCRs in total blood leukocytes, the effect on T and NKT-like cells was not determined (33). It is clear further research is needed specifically on the effect of ROS on T cell and NKT-like cell biology (32).

## Future Therapy for COPD

Lymphocyte senescence and glucocorticoid resistance have been described in several other inflammatory conditions such as cardiovascular disease (34), autoimmune disease (35), arthritis (36), IBD (37) associated with aging (38), and aging with associated inflammation in COPD (39). Some of these conditions are associated with respiratory muscle dysfunction resulting in further increases in ROS and oxidative stress (40). CD28^null^ T cells have been reported in patients with asthma (41), another inflammatory lung disease also associated with increased ROS and oxidative stress (42). Interestingly, several of these inflammatory diseases are also comorbid conditions associated with COPD (10) and therefore may also be associated with increased cytotoxic/pro-inflammatory CD8^+^CD28^null^ NKT-like cells. Hence, targeting the pro-inflammatory nature of these cells by decreasing the expression of Pgp1 and/or CD137 and increasing the expression of GCR, HDAC2, and Hsp90 by CD8^+^CD28^null^ NKT-like cells may reduce inflammation (Figure 2K) associated with a range of steroid resistant diseases including COPD and comorbid conditions associated with COPD. Furthermore, targeting these cytotoxic/pro-inflammatory cells at early onset of COPD may prevent the inevitable spiral of worsening lung function, and associated comorbidity of this progressive debilitating disease, and reduce the associated massive health-care costs (43).

## Author Contributions

GH and SH organized, wrote, and edited the manuscript. Figures were drawn by GH and edited by SH.

## Conflict of Interest Statement

The authors declare that the research was conducted in the absence of any commercial or financial relationships that could be construed as a potential conflict of interest.

## References

[1] Arosa FA CD28+CD28- T cells: certainties and uncertainties of a prevalent human T-cell subset. Immunol Cell Biol (2002) 80:1–13. 10.1046/j.1440-1711.2002.01057 11869357

[2] Fiorentini S Licenzieta S Alessandri G Castelli F Caligaris S Bonafede M CD11b expression identifies CD8+CD28+ T lymphocytes with phenotype and function of both naïve/memory and effector cells. J Immunol (2001) 166(2):900–7. 10.4049/jimmunol.166.2.900 11145666

[3] Urbanowicz RA Lamb JR Todd I Corne JM Fiarclough LC. Altered effector function of peripheral cytotoxic cells in COPD. Respir Res (2009) 10:53. 10.1186/1465-9921-11-76 19545425 PMC2705911

[4] Hodge G Mukaro V Holmes M Reynolds PN Hodge S Enhanced cytotoxic function of natural killer T-like cells associated with decreased CD94 (Kp43) in the chronic obstructive pulmonary disease pathway. Respirology (2013) 18(2):369–76. 10.1111/j.1440-1843.2012.02287 23062183

[5] Tang Y Xiaodan L Wang M Zou Q Zhao S Bowen S Increased numbers of NK cells, NKT-like cells and NK inhibitory receptors in peripheral blood of patients with chronic obstructive pulmonary disease. Clin Dev Immunol (2013) 2013:721782. 10.1155/2013/721782 24069043 PMC3773417

[6] Freeman CM Stolberg VR Crudgington S Martinez FJ Han MK Chensue SW Human CD56+ cytotoxic lung lymphocytes kill autologous lung cells in chronic obstructive pulmonary disease. PLoS One (2014) 9(7):e103840. 10.1371/journal.pone.0103840 25078269 PMC4117545

[7] Tarazona R DelaRosa O Alonso C Ostos B Espejo J Pena J Increased expression of NK cell markers on T lymphocytes in aging and chronic activation of the immune system reflects the accumulation of effector/senescent T cells. Mech Ageing Dev (2001) 121:77–88. 10.1016/j.dci.2008.06.003 11164462

[8] Hodge G Jersmann H Tran HB Holmes M Reynolds PN Hodge S. Lymphocyte senescence in COPD is associated with loss of glucocorticoid receptor expression by pro-inflammatory/cytotoxic lymphocytes. Respir Res (2015) 16:2. 10.1186/s12931-014-0161-7 25573300 PMC4301939

[9] Hodge G Hodge S Li-Liew C Reynolds PN Holmes M Increased natural killer T-like cells are a major source of pro-inflammatory cytokines and granzymes in lung transplant patients. Respirology (2012) 17(1):155–63. 10.1111/j.1440-1843.2011.02075 21995313

[10] Barnes PJ. Glucocorticosteroids: current and future directions. Br J Pharmacol (2011) 163:29–43. 10.1111/j.1476-5381.2010.01199 21198556 PMC3085866

[11] Maeno T Houghton AM Quintero PA Grumelli S Owen CA Shapiro SD. CD8+ T cells are required for inflammation and destruction in cigarette smoke-induced emphysema in mice. J Immunol (2007) 178(2):8090–6. 10.1186/1465-9921-14-13 17548647

[12] Burge PS Calverley PM Jones PW Spencer S Anderson JA Maslen TK. Randomised, doubleblind, placebo controlled study of fluticasone propionate in patients with moderate to severe chronic obstructive pulmonary disease: the ISOLDE trial. BMJ (2000) 320(7245):1297–303. 10.1136/bmj.320.7245.1297 10807619 PMC27372

[13] Celli BR Thomas NE Anderson JA Ferguson GT Jenkins CR Jones PW Effect of pharmacotherapy on rate of decline of lung function in chronic obstructive pulmonary disease. Am J Respir Crit Care Med (2008) 178:332–8. 10.1164/rccm.200712-1869OC 18511702

[14] Hodge G Nairne J Holmes M Reynolds PN Hodge S Increased intracellular T helper 1 pro-inflammatory cytokine production in peripheral blood, bronchoalveolar lavage and intraepithelial T cells of COPD patients. Clin Exp Immunol (2007) 150:22–9. 10.1111/j.1365-2249.2007.03451.x 17614970 PMC2219288

[15] Saetta M Baraldo S Corbino L Turato G Braccioni F Rea F CD8+ve cells in the lungs of smokers with chronic obstructive pulmonary disease. Am J Respir Crit Care Med (1999) 160(2):711–7. 10.1164/ajrccm.160.2.9812020 10430750

[16] Kaur M Smyth LJC Cadden P Grundy S Ray D Plumb J T lymphocyte insensitivity to corticosteroids in chronic obstructive pulmonary disease. Respir Res (2012) 13:20. 10.1186/1465-9921-13-20 22417244 PMC3320534

[17] Fojo AT Ueda K Slamon DJ Poplack DG Gottesman MM Expression of a multidrug resistant gene in human tumors and tissues. Proc Natl Acad Sci U S A (1987) 84:265–9. 10.1073/pnas.84.1.265 2432605 PMC304184

[18] Hodge G Holmes M Jersmann H Reynolds PN Hodge S. The drug efflux pump Pgp1 in pro-inflammatory lymphocytes is a target for novel treatment strategies in COPD. Respir Res (2013) 14:63. 10.1186/1465-9921-14-63 23731729 PMC3681551

[19] Hodge G Jersmann H Tran HB Roscioli E Holmes M Reynolds PN Lymphocyte senescence in COPD is associated with decreased histone deacetylase 2 expression by pro-inflammatory lymphocytes. Respir Res (2015) 16:130. 10.1186/s12931-015-0287-2 26498345 PMC4619495

[20] Barnes PJ Theophylline for COPD. Thorax (2006) 61(9):742–4. 10.1136/thx.2006.061002 16936233 PMC2117081

[21] Xu L Massaque J Nuclear-cytoplasmic shuttling of signal transducers. Nat Rev Mol Cell Biol (2004) 5(3):209–19. 10.1038/nrm1331 14991001

[22] Hodge G Roscioli E Jersmann H Tran HB Holmes M Reynolds PN Steroid resistance in COPD is associated with impaired molecular chaperone Hsp90 expression by pro-inflammatory lymphocytes. Respir Res (2016) 17(1):1–12. 10.1186/s12931-016-0450-4 27769261 PMC5075183

[23] Hoffmann K Handschumacher RE Cycophilin-40: evidence for a dimeric complex with hsp90. Biochem J (1995) 5:8 10.1379/CSC-26R.1 PMC1136737 7717993

[24] Hodge G Mukaro V Reynolds PN Hodge S Role of CD8/CD28(null) T cells and alternate co-stimulatory molecules in chronic obstructive pulmonary disease. Clin Exp Immunol (2011) 166(1):94–102. 10.1111/j.1365-2249.2011.04455 21910726 PMC3193924

[25] Jones D Halting disease in its tracks. Nat Rev Drug Discov (2004) 3:909 10.1038/nrd1692

[26] Hodge G Holmes M Jersmann H Reynolds PN Hodge S. Targeting peripheral blood pro-inflammatory cytotoxic lymphocytes by inhibiting CD137 expression: novel potential treatment for COPD. BMC Pulm Med (2014) 14:85. 10.1186/1471-2466-14-85 24885856 PMC4059030

[27] Rahman I Kinnula V. Strategies to decrease ongoing oxidant burden in chronic obstructive pulmonary disease. Expert Rev Clin Pharmacol (2012) 5(3):293–309. 10.1586/ecp.12.16 22697592 PMC3376391

[28] MacNee W. Pathogenesis of chronic obstructive pulmonary disease. Proc Am Thorac Soc (2005) 2:258–66. 10.1513/pats.200504-045SR 16267346 PMC2713323

[29] Bernardo I Bozinovski S Vlahos R. Targeting oxidant-dependent mechanisms for the treatment of COPD and its comorbidities. Pharmcol Ther (2015) 155:60–79. 10.1016/j.pharmthera.2015.08.005 26297673

[30] Kesarwani P Murali A Al-Khami A Mehrota S. Redox regulation of T-cell function: from molecular mechanisms to significance in human health and disease. Antioxid Redox Signal (2013) 18(12):1497–523. 10.1089/ars.2011.4073 22938635 PMC3603502

[31] Wiley L Ashok D Martin-Ruiz C Talbot DCS Collerton J Kingston A Reactive oxygen species production and mitochondrial dysfunction in white blood cells are not valid biomarkers of aging in the very old. PLoS One (2014) 9(3):e91005 10.1371/journal.pone.0091005 24614678 PMC3948743

[32] Belikov AV Schraven B Simeoni L. T cells and reactive oxygen species. J Biomed Sci (2015) 22:85. 10.1186/s12929-015-0194-3 26471060 PMC4608155

[33] Zeng M Li Y Jiang Y Lu G Huang X Guan K. Local and systemic oxidative stress status in chronic obstructive pulmonary disease patients. Can Respir J (2013) 20(1):35–41. 10.1155/2013/985382 23457673 PMC3628645

[34] Teo FH de Oliveira RT Mamoni RL Ferreira MC Nadruz W Coelho OR Characterisation of CD4+CD28null T cells in patients with coronary artery disease and individuals with risk factors for atherosclerosis. Cell Immunol (2013) 281:11–9. 10.1016/j.cellimm.2013.01.007 23416719

[35] Thewissen M Somers V Hellings N Fraussen J Damoiseaux J Stinissen P CD4+CD28null T cells in autoimmune disease: pathologenic features and decreased susceptibility to immunoregulation. J Immunol (2007) 179(10):6514–23. 10.4049/jimmunol.179.10.6514 17982040

[36] Fasth AE Snir O Johansson AA Nordmark B Rahbar A Klint E Skewed distribution of pro-inflammatory CD4+CD28null T cells in rheumatoid arthritis. Arthritis Res Ther (2007) 9(5):R87 10.1186/ar2286 17825098 PMC2212553

[37] Yokoyama Y Fukunaga K Ikeuchi H Hamikozuru K Hida N Ohda Y The CD4CD28null and the regulatory CD4+CD25High T-cell phenotypes in patients with ulcerative colitis during active and quiescent disease, following colectomy. Cytokine (2011) 56(2):466–70. 10.1016/j.cyto.2011.06.021 21802311

[38] Vallejo AN. CD28 extinction in human T cells: altered functions and the program of T-cell senescence. Immunol Rev (2005) 205:158–69. 10.1111/j.0105-2896.2005.00256.x 15882352

[39] Yao H Rahman I. Role of histone deacetylase 2 in epigenetics and cellular senescence: implications in lung inflammaging and COPD. Am J Physiol Lung Cell Mol Physiol (2012) 303:557–66. 10.1152/ajplung.00175.2012 22842217 PMC3469586

[40] Zuo L Hallman AH Yousif MK Chien MT Oxidative stress, respiratory muscle dysfunction, and potential therapeutics in chronic obstructive pulmonary disease. Front Biol (2012) 7:506–13. 10.1007/s11515-012-1251-x

[41] Hodge S Hodge G Simpson JL Yang IA Upham J James A Blood cytotoxic/inflammatory mediators in non-eosinophilic asthma. Clin Exp Immunol (2016) 46:60–70. 10.1111/cea.12634 26767492

[42] Jiang L Diaz PT Best TM Stimpfl JN He F Zuo L. Molecular characterization of redox mechanisms in allergic asthma. Ann Allergy Asthma Immunol (2014) 113(2):137–42. 10.1016/j.anai.2014.05.030 24986036

[43] Vermeire P. The burden of chronic obstructive pulmonary disease. Respir Med (2002) 96(Suppl C):S3–10. 10.1016/S0954-6111(02)80028-2 12199489

